# Renal Heme Oxygenase-1 Induction with Hemin Augments Renal Hemodynamics, Renal Autoregulation, and Excretory Function

**DOI:** 10.1155/2012/189512

**Published:** 2012-02-08

**Authors:** Fady T. Botros, Leszek Dobrowolski, L. Gabriel Navar

**Affiliations:** ^1^Department of Physiology, Hypertension and Renal Center, Tulane University Health Sciences Center, 1430 Tulane Avenue, SL39, New Orleans, LA 70112, USA; ^2^Laboratory of Renal and Body Fluid Physiology, Mossakowski Medical Research Centre, Polish Academy of Sciences, A. Pawińskiego 5 Street, 02-106 Warsaw, Poland

## Abstract

Heme oxygenases (HO-1; HO-2) catalyze conversion of heme to free iron, carbon monoxide, and biliverdin/bilirubin. To determine the effects of renal HO-1 induction on blood pressure and renal function, normal control rats (*n* = 7) and hemin-treated rats (*n* = 6) were studied. Renal clearance studies were performed on anesthetized rats to assess renal function; renal blood flow (RBF) was measured using a transonic flow probe placed around the left renal artery. Hemin treatment significantly induced renal HO-1. Mean arterial pressure and heart rate were not different (115 ± 5 mmHg versus 112 ± 4 mmHg and 331 ± 16 versus 346 ± 10 bpm). However, RBF was significantly higher (9.1 ± 0.8 versus 7.0 ± 0.5 mL/min/g, *P* < 0.05), and renal vascular resistance was significantly lower (13.0 ± 0.9 versus 16.6 ± 1.4 [mmHg/(mL/min/g)], *P* < 0.05). Likewise, glomerular filtration rate was significantly elevated (1.4 ± 0.2 versus 1.0 ± 0.1 mL/min/g, *P* < 0.05), and urine flow and sodium excretion were also higher (18.9 ± 3.9 versus 8.2 ± 1.0 *μ*L/min/g, *P* < 0.05 and 1.9 ± 0.6 versus 0.2 ± 0.1 *μ*mol/min/g, *P* < 0.05, resp.). The plateau of the autoregulation relationship was elevated, and renal vascular responses to acute angiotensin II infusion were attenuated in hemin-treated rats reflecting the vasodilatory effect of HO-1 induction. We conclude that renal HO-1 induction augments renal function which may contribute to the antihypertensive effects of HO-1 induction observed in hypertension models.

## 1. Introduction

Heme metabolism is catalyzed by heme oxygenases (HO), which convert heme to carbon monoxide (CO), biliverdin, and free iron [1]. Two isoforms of HO are expressed in the kidney, HO-1 and HO-2 [2, 3]. The kidney has relatively low basal level of HO activity [2, 4] that is mainly derived from the constitutive HO-2 [5–7]. Renal HO-2 is localized to epithelial cells of the proximal tubule, thick ascending limb and distal tubule, connecting tubule, and principal cells of the collecting ducts [8]. Renal HO-1 is induced under certain pathological conditions and in response to several agents [3, 9–11]. The pattern of HO-1 expression in the kidney varies with different inducers utilized [2, 12]. Acute treatment with hemin increases renal cortical dialysate CO concentration and causes diuresis and natriuresis [13]. Increases in renal perfusion pressure (RPP) induce renal CO production, and HO inhibition prevents the pressure-dependent increase in CO and attenuates pressure natriuresis [14]. This suggests that induction of HO-1 could modulate renal hemodynamics and renal excretory function.

HO inhibition during nitric oxide synthesis inhibition constricts afferent arterioles (Aff-Art) [15], and exogenous CO administration dilates renal Aff-Art from normal rats [15, 16]. Furthermore, endogenously produced CO exerts a vasodilatory influence on the renal circulation, and inhibition of HO decreases renal blood flow (RBF) [17–21]. Bilirubin is also produced from heme metabolism by HO and biliverdin reductase and is an abundant endogenous antioxidant [22]. Bilirubin scavenges reactive oxygen species [23–25] and inhibits angiotensin II-mediated activation of NADPH oxidase [26, 27], effects that also potentially cause dilation of the renal microvasculature. Chronic treatment of angiotensin-II-infused hypertensive rats with bilirubin attenuates urinary protein excretion [28], and inhibition of bilirubin metabolism attenuates angiotensin II-dependent hypertension in mice [29]. Upregulation of renal HO-1 increases CO and biliverdin/bilirubin production, reduces NADPH oxidase-mediated oxidative stress [26], inhibits cortical 20-HETE synthesis [30], and inhibits thromboxane synthase [31]. Overexpression of HO-1 reduces the pressor responsiveness to angiotensin II [32], and inhibition of HO activity magnifies the renal vasoconstrictor effect of angiotensin II and enhances pressure-induced constriction of isolated pressurized renal interlobular arteries [20]. These results implicate HO-derived metabolites as important modulators of renal microcirculatory function. However, recent data indicate that blood pressure and RBF responses to increased angiotensin II or inhibition of nitric oxide are not significantly enhanced in HO-2 knockout mice [33].

This study was designed to examine the hypothesis that renal HO-1 induction augments renal hemodynamics and renal excretory function. To test this hypothesis, we performed renal clearance and renal hemodynamic studies on control and hemin-treated rats. Accordingly, the aims of this study were (1) to determine the effects of HO-1 induction on renal blood flow (RBF), glomerular filtration rate (GFR), and renal excretory function and (2) to determine the effects of HO-1 induction on RBF autoregulatory responses to changes in RPP and on the renal vasoconstrictor responses to angiotensin II.

## 2. Methods

### 2.1. Animal Treatment

All experimental protocols were approved by Tulane Institutional Animal Care and Use Committee. Male Sprague Dawley rats weighing 300–400 g were fed a normal rat diet (TD 90229, Harlan-Teklad) with free access to water. Two groups of rats were studied: (1) control rats and (2) Hemin-treated rats which received 4 i.p. injections of hemin for 4 consecutive days (3 mg/100 g Bwt./day) prior to the acute experiment.

### 2.2. Renal Function Studies

As described by Patterson et al. [34] and Wang et al. [35], on the day of acute experiment, rats were anesthetized with inactin (thiobutabarbital sodium, Sigma, Saint Louis, USA, 100 mg/kg, i.p.) and placed on thermostatically controlled heated surgical table to maintain rectal temperature at 37°C. A polyethylene tube was placed in the trachea and the animals were allowed to breathe air enriched with oxygen (95% O_2_/5% CO_2_). The left carotid artery was cannulated to allow continuous monitoring of systemic arterial blood pressure and heart rate. The left femoral vein was cannulated to allow infusion of solutions. The left kidney was exposed from a flank incision, gently freed, and placed in a plastic cup. For timed urine collections, a catheter was introduced into the ureter and passed to the pelvis. The renal artery was separated carefully from the renal vein which enabled placement of a noncannulating flow probe, 1 mm in the diameter, connected with a Transonic flowmeter (Transonic System Inc., Ithaca, NY, USA) for measurement of the total RBF. During surgery, an isotonic saline solution containing albumin (6 g/dL) was infused at 1.2 mL/h (for 300 g body weight). Following the surgical procedures, an isotonic saline solution containing 1% albumin and 7.5% polyfructosan (Inutest, Fresenius Kabi, Austria) was infused; initially a priming dose was infused at the rate of 1.6 mL/kg for 5 min followed by a continuous infusion at 1.2 mL/h and a stabilization period of about 1 h was allowed.

#### 2.2.1. Experimental Protocol

**Protocol 1**

experiments were performed to determine the effects of HO-1 induction with hemin on renal hemodynamics and renal excretory function. After the stabilization period, at least six 15-minute collection periods were initiated during which measurements of mean arterial pressure (MAP), heart rate (HR), RBF, renal vascular resistance (RVR), GFR, urine flow (UV), and urinary sodium excretion (U_Na_V) were averaged. Measurements for all collection periods were then averaged. The data from control and hemin-treated rats were compared.

**Protocol 2**

these experiments were done to determine the effects of HO-1 induction with hemin on RBF autoregulatory responses. An aortic clamp was placed above the junction of the left renal artery to regulate RPP to the left kidney. The left femoral artery was cannulated, and the catheter was advanced up the aorta to measure RPP at the left kidney. A Transonic flow probe placed around the left renal artery was used to measure changes in RBF in response to decreases in RPP. After the stabilization period, RPP was decreased to 110, 100, 90, and 80 mmHg and was maintained for 5 minutes at each level. MAP, HR, RBF, and RPP were averaged for each time period at different RPP.

**Protocol 3**

these experiments were done to determine the effects of HO-1 induction with hemin on renal vascular responses to angiotensin II. After the stabilization period, MAP and RBF were measured during i.v. infusion of vehicle followed by angiotensin II (Ang II 50, 100, and 200 ng/kg/min). Each infusion period lasted 30 minutes, and RBF responses were averaged during the last 20 minutes.

### 2.3. Analytical Procedures and Hemin Preparation

Urine volumes were determined gravimetrically. Blood samples were collected in heparinized tubes and centrifuged at 2500 g for 10 min at 4°C to separate the plasma, which was stored at −20°C. Plasma and urine sodium concentrations were measured by flame photometry. Polyfructosan concentrations in urine and plasma samples were measured by standard spectrophotometry. GFR was determined from the clearance of polyfructosan.

Hemin (Sigma-Aldrich, St. Louis, MO): 50 mg hemin was dissolved in 1 mL of 0.1 M NaOH; this solution was diluted using deionized water and pH was adjusted to 7.8; the final volume was adjusted to 5 mL.

#### 2.3.1. Tissue Preparation

Left kidneys were immediately collected and frozen at −80°C until used for Western blot. Tissues were homogenized in buffer at pH 7.4, containing 0.25 M sucrose. The homogenates were centrifuged in an Eppendorf centrifuge at 10,000 g for 10 min at 4°C to remove unbroken cells and the supernatant was stored at −80°C. Protein concentration was determined according to the method of Bradford (BioRad, Hercules, CA) [36].

#### 2.3.2. Western Blot Analysis

As previously described [30], cell-free homogenates (10,000 ×g supernatant) of kidney preparations were separated by SDS/polyacrylamide gel electrophoresis and transferred to a hydrophobic polyvinylidene difluoride** (**PVDF) transfer membrane (Amersham-GE Biosciences, Piscataway, NJ). The membranes were incubated with Odyssey blocking reagent (LI-COR Biosciences, Lincoln, Nebraska) at 4°C overnight. The membranes were incubated for 1 hr with one of the following antibodies: rabbit anti-rat HO-1 and HO-2 polyclonal antibodies (1 : 1000, Stressgen Biotechnologies Corp, Victoria, BC, Canada) or mouse anti-*β*-actin monoclonal antibody. The membranes were washed with phosphate buffered saline tween-20 (PBST) and subsequently probed with fluorescent tagged secondary antibodies at a dilution of 1 : 15000. The signal was detected using Odyssey fluorescent scanner.

### 2.4. Statistical Analysis

Results are presented as mean ± SE for a number (*n*) of experiments. Unpaired *t*-test was used to analyze differences in basal measurements, and HO-1 and HO-2 protein expression between the control and hemin-treated groups. Repeated measures one-way ANOVA followed by Bonferroni's multiple comparison test was used to analyze changes within the same group. Repeated measures two-way ANOVA followed by Bonferroni's multiple comparison test was used to analyze differences between groups. *P* < 0.05 was considered statistically significant.

## 3. Results

### 3.1. HO-1 Protein Expression Is Induced in Kidneys from Hemin-Treated Rats

**Figure 1:**
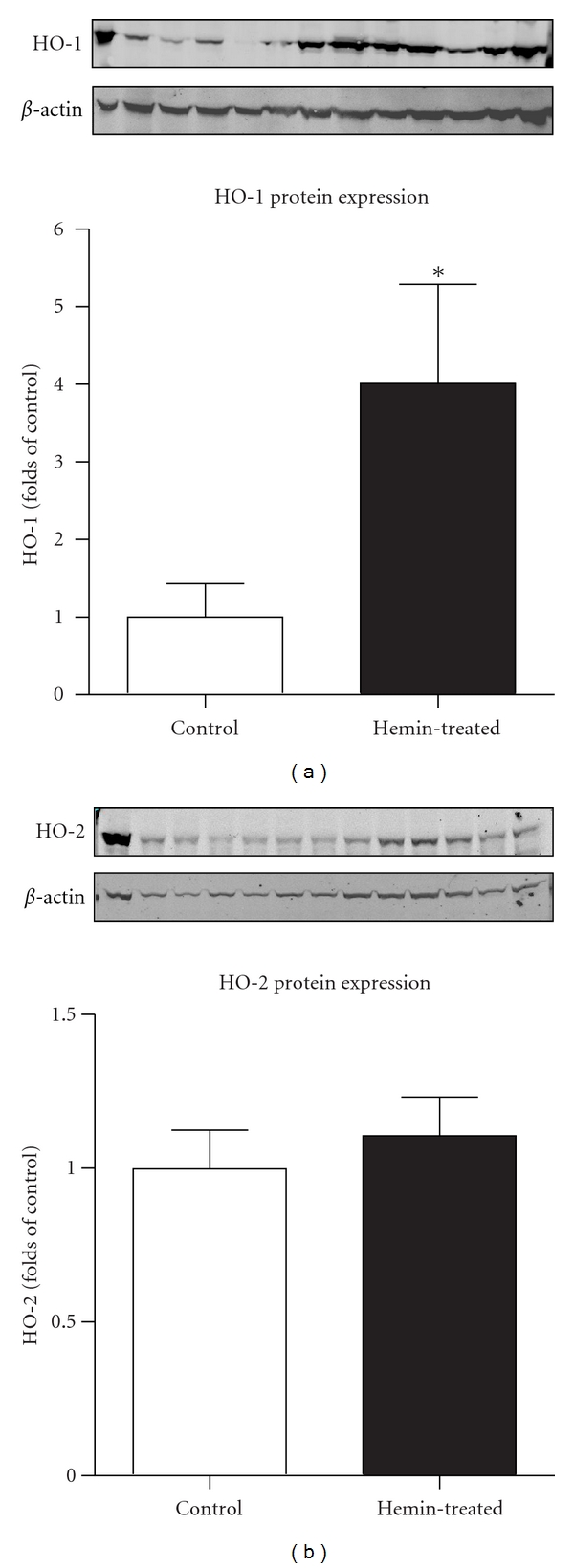
Western blot and densitometry analysis showing the effect of treatment with Hemin (3 mg/100 g body wt/day for 4 days) on renal HO-1 and HO-2 protein expression (*n* = 6) compared to control rats (*n* = 6). Results are normalized by *β*-actin and expressed as folds of control and presented as mean ± SE for each group, *P < 0.05 versus Control.

HO-1 protein expression significantly increased by 4.0 ± 1.3-fold in kidneys from hemin-treated rats compared to kidneys from normal control rats (*n* = 6, *P* < 0.05) (Figure 1). No differences in renal HO-2 expression between hemin-treated rats and normal control rats were detected (Figure 1).

### 3.2. Renal HO-1 Induction with Hemin Is Associated with Augmented Renal Hemodynamics and Excretory Function without Alteration in Mean Arterial Pressure or Heart Rate

**Figure 2:**
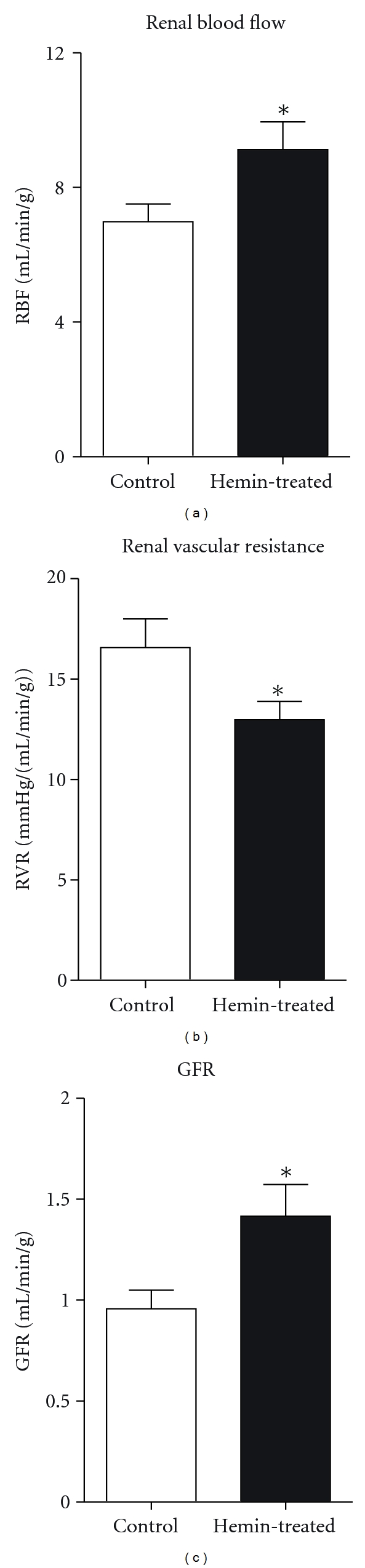
Renal blood flow (RBF), renal vascular resistance (RVR), and glomerular filtration rate (GFR) in hemin-treated rats (*n* = 6) compared to control rats (*n* = 7). Results are expressed as mean ± SE for each group, *P < 0.05 versus Control.

HR and MAP were not significantly different in hemin-treated rats compared to control rats (Table 1). While RBF and GFR were significantly higher, RVR was significantly lower in hemin-treated rats (Table 1 and Figure 2). These data indicate that renal HO-1induction is associated with renal vasodilation in hemin-treated rats.

**Figure 3:**
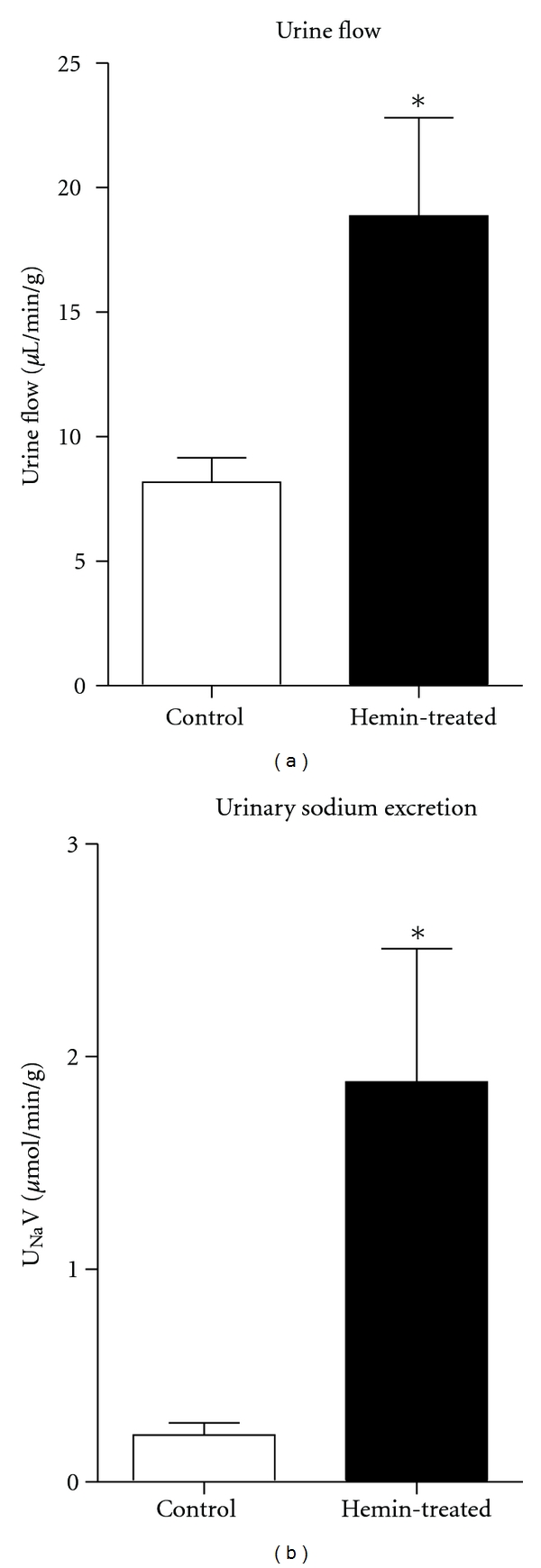
Urine flow (UV) and urinary sodium excretion (U_Na_V) in hemin-treated rats (*n* = 6) compared to control rats (*n* = 7). Results are expressed as mean ± SE for each group, *P < 0.05 versus Control.

Hemin-treated rats also had increased urine flow and urinary sodium excretion (U_Na_V) indicating that renal HO-1 induction is associated with augmented renal excretory function (Table 1 and Figure 3).

### 3.3. Hemin-Treatment Increases the RBF Autoregulatory Plateau in Response to Decreases in RPP

**Figure 4:**
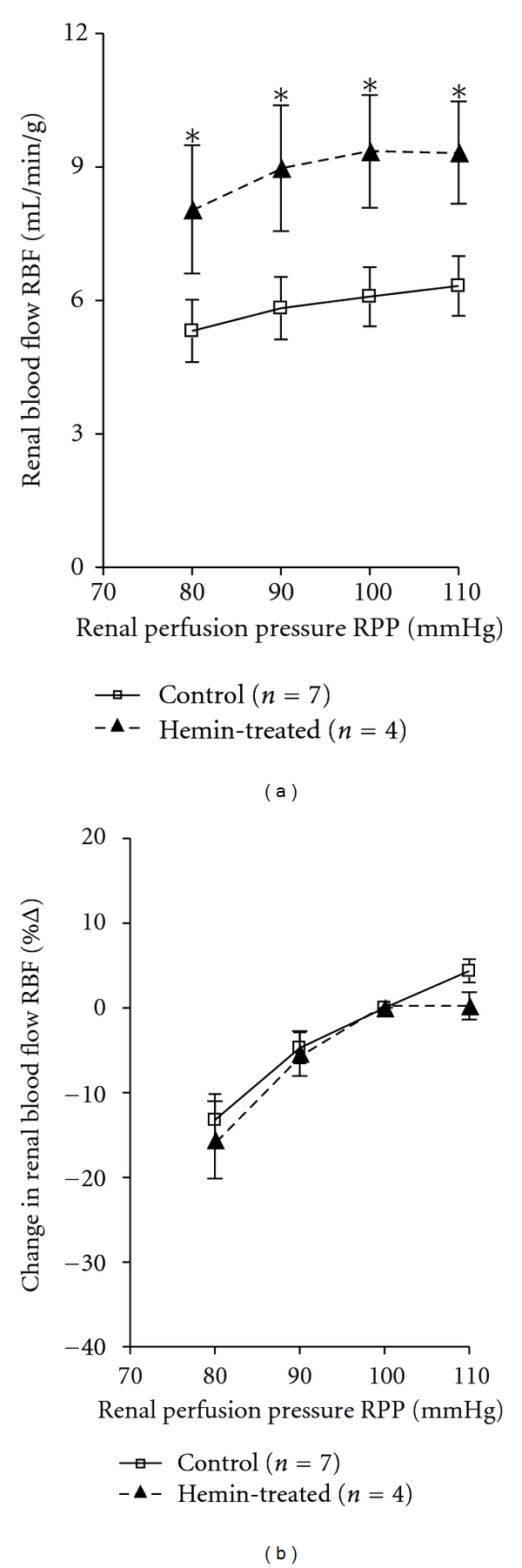
Renal blood flow (RBF) autoregulatory responses to changes in renal perfusion pressure (RPP) in hemin-treated rats (*n* = 4) compared to control rats (*n* = 7). Results are expressed as mean ± SE for each group, *P < 0.05 versus Control.

Figure 4(a) shows that RBF was significantly higher in hemin-treated rats compared to control rats at all measured RPP points indicating that the vasodilatory effect is maintained at different renal perfusion pressures.  Figure 4(b) demonstrates that RBF autoregulatory responses to changes in RPP were not altered in hemin-treated rats indicating that renal autoregulatory responses are maintained with a tendency to show improved autoregulatory capability.

### 3.4. Renal HO-1 Induction with Hemin Is Associated with Attenuated Renal Vasoconstrictor Responses to Ang II and Maintained Pressor Responses

Basal MAP was not significantly different between control and hemin treated rats (126.0 ± 2.1 mmHg, *n* = 7 versus 118.9 ± 3.9 mmHg, *n* = 5). RBF was significantly higher in hemin treated rats compared to control rats (10.5 ± 0.7 mL/min/g, *n* = 5 versus 8.1 ± 0.9 mL/min/g, *n* = 7, *P* < 0.05).

**Figure 5:**
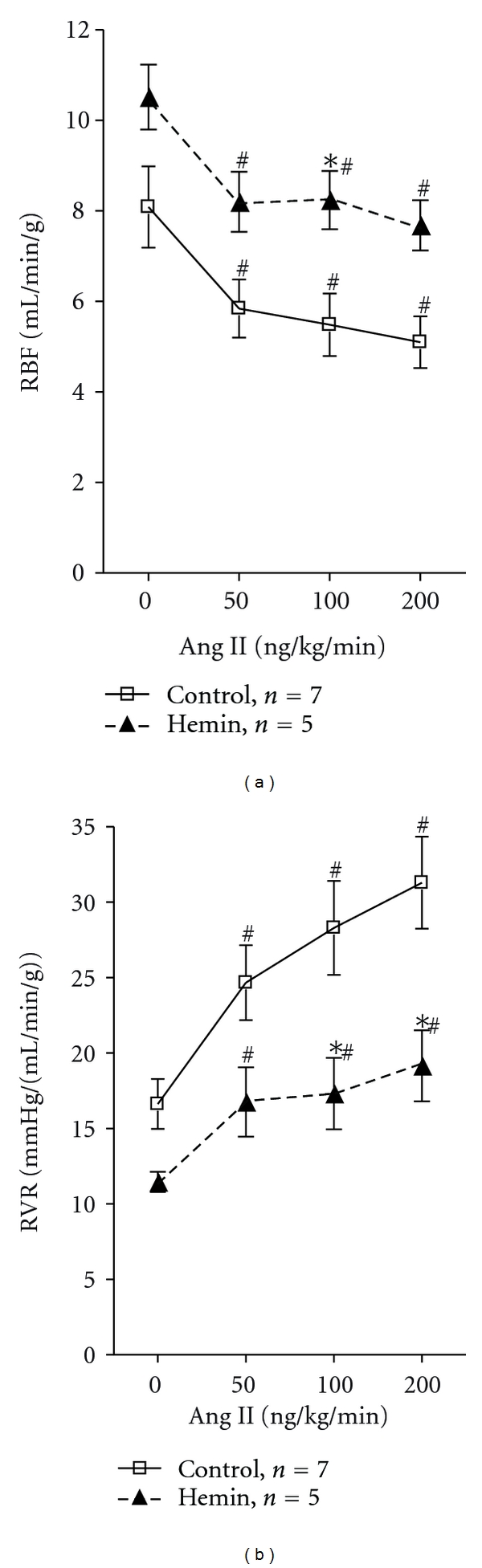
Changes in RBF and RVR in response to infusion of angiotensin II in hemin-treated rats (*n* = 5) compared to control rats (*n* = 7). Results are expressed as mean ± SE for each group, *P < 0.05 versus Control, ^#^ *P* < 0.05 versus baseline before angiotensin II infusion.

In response to angiotensin II infusion, MAP significantly increased to the same extent in both groups. In response to infusion with Ang II 50, 100, and 200 ng/kg/min, MAP increased from 126.0 ± 2.1 mmHg to 134.9 ± 1.7, 142.7 ± 1.6, and 150.7 ± 2.2 mmHg in the control group (*n* = 7) and from 118.9 ± 3.9 to 131.4 ± 7.2, 136.9 ± 9.3, and 142.7 ± 9.7 mmHg in the hemin-treated rats (*n* = 5). RBF significantly decreased in response to infusion with Ang II 50, 100, and 200 ng/min/kg in both groups; however, RBF was significantly higher in the hemin-treated group compared to the control group (*P* < 0.0001). RBF during infusion with 200 ng/min/kg Ang II was 7.7 ± 0.6 mL/min/g in hemin-treated group (*n* = 5) compared to 5.1 ± 0.6 mL/min/g in control group (*n* = 7) (Figure 5). The reduced responsiveness to Ang II infusion in the hemin treated group is shown by the attenuated increases in RVR with the Ang II infusions.

## 4. Discussion

HO-1 plays a major renoprotective role when induced under certain pathophysiological conditions [4, 9, 17, 19]. Although several reports examined effects of acute activation or inhibition of HO via acute administration of the substrate hemin or HO inhibitors, the effects of chronic induction of HO-1 on renal hemodynamics and renal excretory function have not been examined before. To determine the effects of HO-1 on renal function, hemin was used to induce HO-1 expression [1]. Hemin increases HO activity via increasing substrate availability and also by transcriptional activation of hmox-1 gene [37]. Treating with HO-1 inducers causes differential induction of HO-1 in the kidney; increased expression of HO-1 in different renal structures may exert different effects on renal function [38]. As expected, in our study chronic hemin administration increased HO-1 expression whereas it did not affect HO-2 expression.

In rats treated with hemin, MAP was not different compared to untreated rats; however, hemin-treated rats had significantly higher RBF and lower RVR. These results indicate that renal HO-1 induction exerts a renal vasodilatory effect. This is consistent with previously reported data that acute heme administration increases RBF, urine flow, and sodium excretion [13]. In addition, hemin-treated rats had significantly higher GFR compared to control rats indicating that the vasodilatory response is mediated through dilation of renal arterioles. These data are consistent with previous reports showing that acute inhibition of HO decreases RBF [18, 20] and infusion of CO increased RBF and GFR. In addition, CO causes vasodilation of afferent arterioles [15]. Interestingly, this renal vasodilatory response in hemin-treated rats was associated with significantly higher urine flow (2-fold) and sodium excretion (10-fold) (Figure 3), indicating that HO-1 induction exerts diuretic and natriuretic responses. This is consistent with previous data showing that acute heme administration causes natriuresis [13], while acute HO inhibition decreases urine flow and sodium excretion [39]. With chronic treatment, induction of HO-1 may be in different segments of the nephron and may be inhibiting other ion transporters; however, the effects of HO products on tubular transporters have not been well characterized. In addition, previous research shows that acute inhibition of HO with CrMP attenuates pressure-natriuresis, indicating that HO participates in the natriuretic response to increases in arterial pressure [14]. We speculate that the effects of chronic HO-1 induction on renal function are mediated directly via increases in CO and/or bilirubin production. However, indirect effects via regulation of other heme enzymes and/or renal transporters cannot be excluded.

To determine the effect of this vasodilatory response on renal autoregulatory capability, renal perfusion pressure was decreased using an aortic clamp and the corresponding RBF was recorded. Data presented in Figure 4 indicate that renal autoregulatory responses are maintained in hemin-treated rats albeit at higher RBF and changes in RBF in response to decreases in RPP were comparable in hemin-treated and control rats. This indicates that the renal vascular responses to changes in RPP are maintained; however, the plateau of the autoregulation relationship is elevated reflecting the vasodilatory effect of HO-1 induction. This may protect the kidney from excessive vasoconstriction during hypertension while limiting the deleterious effects of the increases in renal perfusion pressure, which could explain the renoprotective role of HO induction in different models of hypertension [38]. To examine this hypothesis, changes in RBF in response to acute infusion of angiotensin II were compared between hemin-treated and control untreated rat.

Angiotensin II infusion increased MAP to the same level in hemin-treated rats and control rats; however, renal vasoconstrictor responses were significantly attenuated in hemin-treated rats (Figure 5). This observation is consistent with previous data showing that the inhibition of HO with SnMP magnifies the renal vasoconstrictor effect of angiotensin II [20]. Although RBF significantly decreased in both control and hemin-treated rats, the increase in RVR in response to angiotensin II was significantly greater in control rats than in hemin-treated rats (Figure 5). This finding is of significant importance because induction of HO-1 by heme administration to angiotensin-II-infused hypertensive rats increases creatinine clearance and decreases proteinuria [17], indicating a renoprotective effect of HO-1 during angiotensin II hypertension. In addition, chronic hemin infusions normalize blood pressure in spontaneously hypertensive rats [40] and induction of HO-1 prevents the blood pressure increase in angiotensin-II-infused mice [41].

In summary, the present study demonstrates that pharmacological renal HO-1 induction increases RBF, GFR, urine flow, and sodium excretion; these effects are conducive to renoprotection. Renal autoregulatory responses are maintained but operate at a higher RBF during HO-1 induction. Finally, renal HO-1 induction with hemin significantly attenuates renal vasoconstrictor responses to angiotensin II. Under several pathophysiological conditions, HO-1 is induced in the kidney [9]; renal HO-1 induction under these circumstances might play a renoprotective role by attenuating excessive renal vasoconstrictor responses.

## Figures and Tables

**Table 1:** Hemodynamics and renal excretory measurements in hemin-treated rats compared to control untreated rats.

	Control (*n* = 7)	Hemin (*n* = 6)
Mean arterial pressure, MAP (mmHg)	112 ± 4	115 ± 5
Heart rate, HR (bpm)	346 ± 10	331 ± 16
Glomerular filtration rate, GFR (mL/min/g)	1.0 ± 0.1	1.4 ± 0.2*
Renal blood flow, RBF (mL/min/gm)	7.0 ± 0.5	9.1 ± 0.8*
Renal vascular resistance, RVR [mmHg/(mL/min/g)]	16.6 ± 1.4	13.0 ± 0.9*
Urine flow, UV (*μ*L/min/g)	8.2 ± 1.0	18.9 ± 3.9*
Sodium excretion, U_Na_V (*μ*mol/min/g)	0.2 ± 0.1	1.9 ± 0.6*

*P < 0.05 versus control.
